# Synthesis and biological evaluation of *N*-arylpiperazine derivatives of 4,4-dimethylisoquinoline-1,3(2*H*,4*H*)-dione as potential antiplatelet agents

**DOI:** 10.1080/14756366.2018.1437155

**Published:** 2018-02-27

**Authors:** Monika Marcinkowska, Magdalena Kotańska, Agnieszka Zagórska, Joanna Śniecikowska, Monika Kubacka, Agata Siwek, Adam Bucki, Maciej Pawłowski, Marek Bednarski, Jacek Sapa, Małgorzata Starek, Monika Dąbrowska, Marcin Kołaczkowski

**Affiliations:** a Department of Medicinal Chemistry, Jagiellonian University Medical College, Kraków, Poland;; b Department of Pharmacological Screening, Chair of Pharmacodynamics, Jagiellonian University Medical College, Krakó, Poland;; c Department of Pharmacobiology, Jagiellonian University Medical College, Kraków, Poland;; d Department of Pharmacological Screening, Chair of Pharmacodynamics, Jagiellonian University Medical College, Kraków, Poland;; e Department of Inorganic and Analytical Chemistry, Faculty of Pharmacy, Jagiellonian University Medical College, Krakow, Poland

**Keywords:** Antiplatelet agents, blockade of the platelet aggregation, alpha 2B receptor antagonists, ARC-239

## Abstract

Despite the substantial clinical success of aspirin and clopidogrel in secondary prevention of ischemic stroke, up to 40% of patients remain resistant to the available antiplatelet treatment. Therefore, there is an urgent clinical need to develop novel antiplatelet agents with a novel mechanism of action. Recent studies revealed that potent alpha 2B-adrenergic receptor (alpha 2B-ARs) antagonists could constitute alternative antiplatelet therapy. We have synthesized a series of *N*-arylpiperazine derivatives of 4,4-dimethylisoquinoline-1,3(2*H*,4*H*)-dione as potential alpha 2B receptor antagonists. The most potent compound **3**, effectively inhibited the platelet-aggregation induced both by collagen and ADP/adrenaline with IC_50_ of 26.9 μM and 20.5 μM respectively. Our study confirmed that the alpha 2B-AR antagonists remain an interesting target for the development of novel antiplatelet agents with an alternative mechanism of action.

## Graphical abstract

**Figure:**
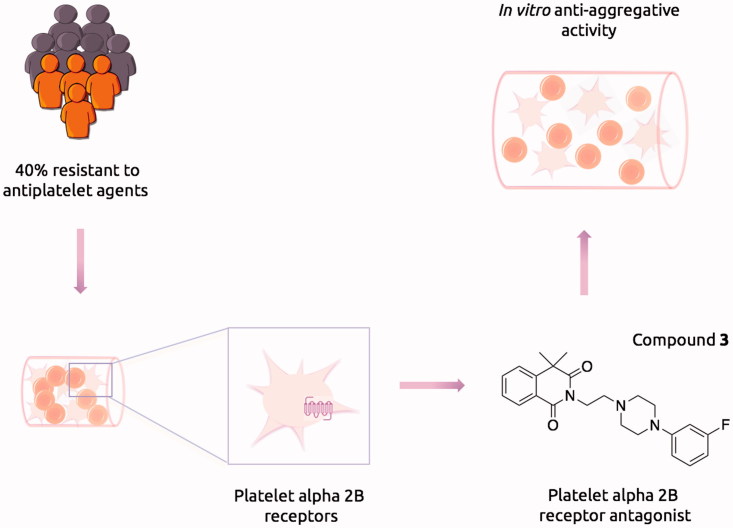

## Introduction

Antiplatelet drugs are the mainstay of the pharmacological treatment for patients with various cardiovascular diseases^1^. Large clinical trials have revealed that treatment with antiplatelet agents such as clopidogrel and aspirin may reduce the risk of myocardial infraction, stroke or death by almost 22%^2^. This fact has made them one of the most widely prescribed drugs in the world^3^. However, despite significant clinical success in preventing the adverse outcome of cardiovascular diseases, many patients experience recurrent atherothrombotic events, despite the treatment with antiplatelet agents. Moreover, many patients are resistant to aspirin and/or clopidogrel, which results in poor prognosis and increased risk of further cardiovascular events^2^.

Clopidogrel and aspirin act via a blockade of adenosine diphosphate (ADP) receptor and inhibition of cyclooxygenase-1 (COX-1) respectively. These mechanisms result in the inhibition of the platelet activation and aggregation and further clot formation^4^. It has been suggested that aspirin resistance may be related to the lack or insufficient inhibition of the COX-1–mediated thromboxane A2 pathway, while clopidogrel resistance is related to the P2Y12 ADP receptor signaling^5^ ^,^ ^6^. Therefore, there is an urgent clinical need to develop novel antiplatelet agents involving different pathways of platelet aggregation, which would constitute an alternative for the treatment of resistant patients.

Recent studies revealed that the blockade of platelet alpha 2B-adrenergic receptors (alpha 2B-ARs) may play a role in platelet aggregation^7^ ^,^ ^8^. Interestingly, inhibition of alpha 2B-ARs in patients with ischemic heart disease, treated with clopidogrel and aspirin, resulted in an additional antiplatelet effect^9^. Moreover, it has been shown that adrenaline under the stimulation of alpha-adrenergic receptors leads to increased platelet aggregation and may overcome the aspirin-induced blockade of platelet function^10^ ^,^ ^11^. Therefore, the blockade of platelet alpha 2B-ARs may have also a clinical benefit for aspirin-resistant patients. The results of these studies suggest that the blockade of platelet alpha 2B-ARs offers a new therapeutic strategy for the development of novel antiplatelet agents.

Among many structurally different classes of alpha adrenergic ligands, arylpiperazine derivatives have been the most intensively investigated^12^. The conformationally rigid arylpiperazine fragment is crucial for proper interactions with the alpha 2B-AR. It provides charge-reinforced hydrogen bond between nitrogen atom of piperazine ring and Asp3.32 residue from the orthosteric binding site of alpha 2B-ARs. At the same time, the phenyl ring enables essential aromatic CH-π stacking with Phe6.52, which provides further stabilization of the ligand-receptor complex, along with interactions in the second (allosteric) binding site (Figure 2)^13^ ^,^ ^14^.

**Figure 1.**
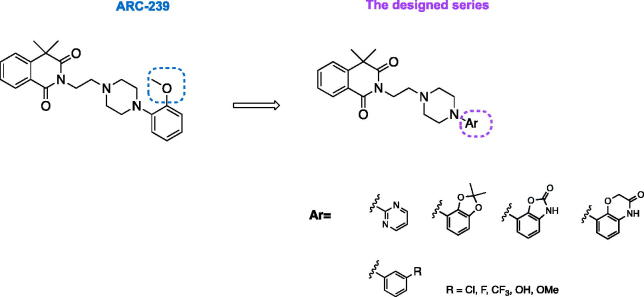
Structures of the designed 4,4-dimethylisoquinoline-1,3(2*H*,4*H*)-dione derivatives.

A phenylpiperazine derivative of 4,4-dimethylisoquinoline-1,3(2*H*,4*H*)-dione, compound ARC-239 (Figure 1), is a well-known, potent alpha 2B receptor antagonist, selective vs. 2 A subtype^15^. However, as an *orto*-methoxyphenylpiperazine derivative, ARC-239 shares a similar pharmacophore with alpha 1 adrenoceptor ligands^12^. In fact, our research, in addition to other literature reports, show that ARC-239 exhibits strong binding affinity also for alpha 1 adrenoreceptor (Table 1)^12^ ^,^ ^16^, which could be the source of additional unwanted adverse reactions^17^. We used the compound ARC-239 as a starting point in the design of selective alpha 2B-AR ligands with antiplatelet activity, assuming that it can be deprived of the alpha 1 adrenergic activity, by changing the substitution pattern at the phenylpiperazine moiety. It has been reported that *ortho*-methoxyphenyl group is privileged for alpha 1 A receptor affinity^18^ ^,^ ^19^. Therefore, in order to obtain selective alpha 2B-AR ligands, we replaced the *ortho*-methoxyphenyl group with meta-substituted phenyl moiety or with bulkier heteroaromatic rings, all acceptable for alpha 2B-AR binding site restrictions, while maintaining the 4,4-dimethylisoquinoline-1,3(2*H*,4*H*)-dione scaffold unchanged (Figure 1). The latter moiety has been recognized as a key pharmacophore fragment and therefore its replacement might result in loss of affinity or selectivity towards alpha adrenergic receptors^20^. Therefore, we postulated that modifications restricted to the phenylpiperazine scaffold would reduce the interaction with alpha 1-AR while maintaining the alpha 2-AR affinity.

**Table 1.** Molecular properties and PAINS analysis.

	Lipinski rule of 5				Veber filter		PAINS
Compound	QPLogP	MW	HBD	HBA	RB	TPSA	#Alerts
**3**	4.1	395.5	0	6	3	55.3	0
**4**	4.4	411.9	0	6	3	55.4	0
**5**	5.0	445.5	0	6	3	59.7	0
**6**	3.9	407.5	0	7	4	63.6	0
**7**	3.3	393.5	1	7	4	78.0	0
**8**	2.7	379.5	0	8	3	73.9	0
**9**	4.1	449.5	0	8	3	70.6	0
**10**	2.4	434.5	1	9	3	109.3	0
**11**	2.8	448.5	1	9	3	104.6	0

Moreover, previous research showed that bulky aromatic substituents rich in π electrons may increase the electrostatic interactions with aromatic amino acid residues of alpha 2-AR binding pocket^21^ ^,^ ^22^. Therefore, by enhancing the electron density with a proper aromatic substituent, we expected to strengthen interactions between the aromatic ring and Phe6.52 residue of alpha 2-AR binding pocket and thus increase ligand affinity for this molecular target.

Furthermore, in order to avoid interaction with other monoaminergic receptors (e.g. serotonin 5-HT1A, 5-HT2A, and dopamine D2), we kept the original ethyl chain, linking the phenylpiperazine fragment and 4,4-dimethylisoquinoline-1,3(2*H*,4*H*)-dione. It is worth mentioning that previously it has been shown that increasing the length of an alkyl linker in phenylpiperazine derivatives might result in increased affinity towards the above-mentioned undesirable receptor targets^23^ ^,^ ^24^.

**Figure 2.**
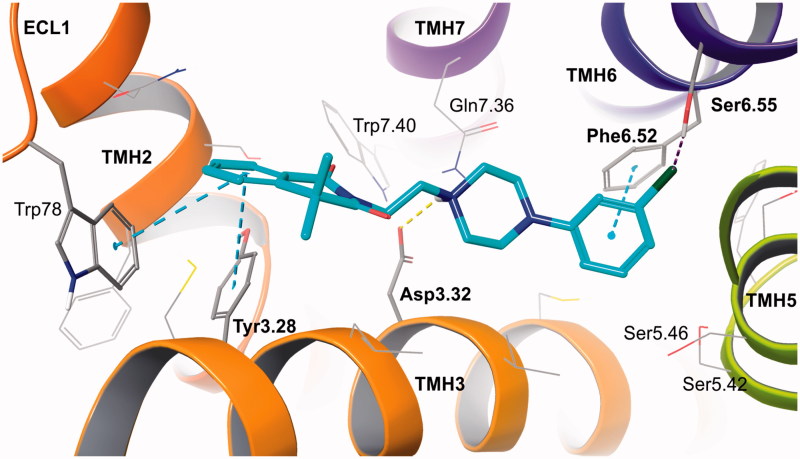
Prototype compound 4 in alpha 2B adrenergic receptor homology model based on beta 2 adrenergic receptor crystal structure (2RH1). Amino acid residues engaged in ligand binding (within 4 Å from the ligand atoms) are displayed as sticks, whereas those forming typical H-bonds (dotted yellow line), π-π stacking (dotted blue lines) or H-bonds to halogens (dotted purple line) are represented as thick sticks. The extracellular loop (ECL) 2 was hidden for clarity. TMH – transmembrane helix.

The proposed binding mode of the designed compounds, presented on the example of the prototype compound **4**, shows well-recognized anchoring interactions of arylpiperazine fragment in the orthosteric binding site, between transmembrane helices (TMHs) 3, 5, and 6^25^ ^,^ ^26^. Those include charge-reinforced hydrogen bond of protonated piperazine with Asp3.32 and π-π stacking of 3-chlorophenyl ring with Phe6.52, additionally stabilized by weak interaction of chlorine substituent with Ser6.55. The standard interactions for monoaminergic receptor ligands are complemented by 4,4-dimethylisoquinoline-1,3(2*H*,4*H*)-dione aromatic bonds (π-π stacking) with residues from the second (allosteric) binding site. These are Tyr3.28 from TMH3 and Trp78 from extracellular loop (ECL) 1 (Figure 2).

## Materials and methods

### Molecular modeling

Ligand docking studies involved human adrenergic alpha 2B receptor homology model, developed using the well-validated method^27^.

The novel homology model was built on the basis of adrenergic β_2_ receptor crystal structure (PDB ID: 2RH1)^28^. Sequence alignment between target receptor (UniProt database accession number P18089) and the template were performed by hhsearch tool via GeneSilico Metaserver (https://www.genesilico.pl/meta2/)^29^. The artificial fragments replacing the third intracellular loop (ICL3) in the protein crystal structure were removed and short loops were created. The crude receptor models were obtained using SwissModel (https://swissmodel.expasy.org/)^30^ and were validated by processing in Protein Preparation Wizard. ARC-239 structure was utilized for ligand-based binding site optimization, performed using induced fit docking (IFD) workflow. That procedure resulted in conformational receptor model that served as molecular target in docking studies.

Ligand structures were optimized using LigPrep tool (Schrödinger, LLC, New York, USA). Glide SP flexible docking procedure was carried out using default parameters. OPLS3 force field was applied on both energy minimization (protein and ligands) and docking stages. H-bond constraint, as well as centroid of a grid box for docking studies were located on Asp3.32.

Molecular properties were calculated using QikProp software (Schrödinger, LLC, New York, USA) (QPLogP – Predicted octanol/water partition coefficient; MW – molecular weight; HBD – hydrogen bond donor; HBA – hydrogen bond acceptor; RB – rotatable bonds; TPSA – total polar surface area). Number of PAINS alerts determined by SwissADME server (http://www.swissadme.ch)^31^, ADME parameters were predicted by: QikProp (QPlogS – solubility; QPPCaco – Caco-2 cell permeability; % PO Absorption – percent human oral absorption), SwissADME (BBB – blood-brain barrier permeability; Pgp – substrate of glycoprotein P) and VolSurf + version 1.0.7.1 from Molecular Discovery (Borehamwood, UK) (PB – % of protein binding; MetStab – metabolic stability after CYP incubation).

Glide, induced fit docking, LigPrep, Protein Preparation Wizard, and QikProp were implemented in Small-Molecule Drug Discovery Suite (Schrödinger Release 2017–1: Maestro, Schrödinger, LLC, New York, NY, USA, 2017), which was licensed for Jagiellonian University Medical College.

### Chemistry

Unless otherwise indicated, all the starting materials and the reference compound ARC-239 were obtained from commercial suppliers and were used without further purification. Analytical thin-layer chromatography (TLC) was performed on Merck Kieselgel 60 F_254_ (0.25 mm) pre-coated aluminum sheets (Merck, Darmstadt, Germany). Visualization was performed with a 254 nm UV lamp. Column chromatography was performed using silica gel (particle size 0.063–0.200 mm; 70–230 Mesh ATM) purchased from Merck. The UPLC-MS or UPLC-MS/MS analyses were run on UPLC-MS/MS system comprising Waters ACQUITY® UPLC^®^ (Waters Corporation, Milford, MA, USA) coupled with Waters TQD mass spectrometer (electrospray ionization mode ESI with tandem quadrupole). Chromatographic separations were carried out using the ACQUITY UPLC BEH (bridged ethyl hybrid) C_18_ column: 2.1 × 100 mm and 1.7 µm particle size. The column was maintained at 40 °C and eluted under gradient conditions using 95% to 0% of eluent A over 10 min, at a flow rate of 0.3 ml/min. Eluent A: water/formic acid (0.1%, *v/v*); eluent B: acetonitrile/formic acid (0.1%, *v/v*). A total of 10 µl of each sample were injected, and chromatograms were recorded using Waters eλ PDA detector. The spectra were analyzed in the range of 200–700 nm with 1.2 nm resolution and at a sampling rate of 20 points/s. MS detection settings of Waters TQD mass spectrometer were as follows: source temperature 150 °C, desolvation temperature 350 °C, desolvation gas flow rate 600 l/h, cone gas flow 100 l/h, capillary potential 3.00 kV, and cone potential 20 V. Nitrogen was used for both nebulizing and drying. The data were obtained in a scan mode ranging from 50 to 1000 *m/z* at 0.5 s intervals; 8 scans were summed up to obtain the final spectrum. Collision activated dissociation (CAD) analyses were carried out with the energy of 20 eV, and all the fragmentations were observed in the source. Consequently, the ion spectra were obtained in the range from 50 to 500 *m/z*. MassLynx V 4.1 software (Waters) was used for data acquisition. Standard solutions (1 mg/ml) of each compound were prepared in a mixture comprising analytical grade acetonitrile/water (1/1, *v/v*). The UPLC/MS purity of all the test compounds and key intermediates was determined to be >95%. ^1^H NMR and ^13 ^C NMR spectra were obtained in a Varian Mercury spectrometer (Varian Inc., Palo Alto, CA, USA), in CDCl_3,_ operating at 300 MHz (^1^H NMR), 75 MHz (^13 ^C NMR). Chemical shifts are reported in terms of *δ* values (ppm) relative to TMS *δ* = 0 (^1^H) as internal standard. The *J* values are expressed in Hertz. Signal multiplicities are represented by the following abbreviations: s (singlet), br.s (broad singlet), d (doublet), dd (doublet of doublets), t (triplet), q (quartet), m (multiplet). Elemental analysis was performed using the VarioEL III – Elementar apparatus (Hanau, Germany).

### General procedure for the synthesis of 2–(2-chloroethyl)-4,4-dimethylisoquinoline-1,3(2H,4H)-dione (2)

A mixture of 4,4-dimethylisoquinoline-1,3(2*H*,4*H*)-dione (2.11 mmol), 1-bromo-2-chloroethane (5.58 mmol), potassium carbonate (8.68 mmol), trimethylamine (3.96 mmol) in acetone (20 ml) was stirred for 72 h at 55 °C. Next, the reaction mixture was cooled to the room temperature, potassium carbonate was filtered off and the solvent was evaporated under the reduced pressure. The crude mixture was purified via column chromatography using n-hexane:DCM:MeOH 40:59.6:0.5 (v/v) as eluent.

Yield 60%, yellow crystalizing oil, ^1^H NMR (300 MHz, CDCl_3_): δ 8.24–8.18 (m, 1H), 7.67–7.60 (m, 1H), 7.48–7.38 (m, 2H), 4.39–4.33 (t, *J* = 6.6 Hz, 2H), 3.76–3.70 (t, *J* = 6.6 Hz, 2H), 1.63 (s, 6H); Formula: C_13_H_14_ClNO_2_; ESI-MS: 252 [M + H]^+^.

### General procedure for the synthesis of the final molecules (3–11)

#### Method C (for compounds 3–6)

A mixture of 2–(2-chloroethyl)-4,4-dimethylisoquinoline-1,3(2*H*,4*H*)-dione (**2**) (0.247 mmol) and corresponding piperazine (0.5 mmol) was stirred at 140 °C for 30 min. After this time, the reaction mixture was cooled to room temperature, EtOAc (4 ml) was added, and the resulted solid was filtered off. The remaining solution was concentrated in vacuum and further purified via column chromatography using n-hexane:Et_2_O:DCM 20:40:40 or DCM:EtOAc:MeOH 69.8:30:0.2 as eluent.

### 2–(2-(4–(3-Fluorophenyl)piperazin-1-yl)ethyl)-4,4-dimethylisoquinoline-1,3(2H,4H)-dione (3)

Yield 41%, pale yellow oil, ^1^H NMR (300 MHz, CDCl_3_): δ 8.24–8.20 (dd, *J* = 1.0 and 7.7 Hz, 1H), 7.68–7.60 (m,1H), 7.49–7.38 (m, 2H), 7.17–7.10 (t, *J* = 7.9 Hz, 1H), 6.86–6.72 (m, 3H), 4.24–4.17 (t, *J* = 6.6 Hz, 2H), 3.16–3.08 (t, *J* = 4.9 Hz, 4H), 2.70–2.62 (m, 6H), 1.63 (s, 6H); ^13 ^C NMR (75 MHz, CDCl_3_): δ; 177.1, 164.1, 163.8, 152.1, 145.1, 135.2, 129.7, 128.3, 127.3, 127.2, 125.1, 118.7, 115.6, 111.8, 55.6, 53.4 (2 C), 48.6 (2 C), 43.6, 37.1, 29.4 (2 C); Formula: C_23_H_26_FN_3_O_2_; ESI-MS: 396 [M + H]^+^.

### 2–(2-(4–(3-Chlorophenyl)piperazin-1-yl)ethyl)-4,4-dimethylisoquinoline-1,3(2H,4H)-dione (4)

Yield 38%, pale yellow oil, ^1^H NMR (300 MHz, CDCl_3_): δ 8.24–8.20 (dd, *J* = 1.0 and 7.7 Hz, 1H), 7.68–7.60 (m, 1H), 7.49–7.38 (m, 2H), 7.17–7.10 (t, *J* = 7.9 Hz, 1H), 6.86–6.72 (m, 3H), 4.24–4.17 (t, *J* = 6.6 Hz, 2H), 3.16–3.08 (t, *J* = 4.9 Hz, 4H), 2.70–2.62 (m, 6H), 1.63 (s, 6H); ^13 ^C NMR (75 MHz, CDCl_3_): δ 177.0, 164.1, 152.0, 145.0, 135.3, 129.2, 128.5, 127.3, 126.3, 125.6, 123.8, 118.3, 114.6, 111.2, 55.4, 53.3 (2 C), 48.8 (2 C), 43.4, 37.2, 29.3 (2 C); Formula: C_23_H_26_ClN_3_O_2_; ESI-MS: 412 [M + H]^+^.

### 4,4-Dimethyl-2–(2-(4–(3-(trifluoromethyl)phenyl)piperazin-1-yl)ethyl)isoquinoline-1,3(2H,4H)-dione (5)

Yield 35%, pale yellow oil, ^1^H NMR (300 MHz, CDCl_3_): δ 8.24–8.20 (dd, *J* = 1.0 and 7.9 Hz, 1H), 7.67–7.59 (m, 1H), 7.48–7.39 (m, 2H), 7.35–7.22 (t, *J* = 8.2 Hz, 1H), 7.09–6.98 (m, 3H), 4.25–4.15 (t, *J* = 6.6 Hz, 2H), 3.20–3.10 (t, *J* = 4.9 Hz, 4H), 2.72–2.64 (m, 6H), 1.63 (s, 6H); ^13 ^C NMR (75 MHz, CDCl_3_): δ 177.1, 164.1, 151.3, 145.0, 133.9, 131.6 (q, *J* = 63.0 and 31.5 Hz), 129.4, 128.8, 127.3, 126.2, 125.1, 123.8, 118.5, 115.5, 111.8, 55.5, 53.4 (2 C), 48.6 (2 C), 43.5, 37.2, 29.4 (2 C); Formula: C_24_H_26_F_3_N_3_O_2_; ESI-MS: 446 [M + H]^+^.

### 2–(2-(4–(3-Methoxyphenyl)piperazin-1-yl)ethyl)-4,4-dimethylisoquinoline-1,3(2H,4H)-dione (6)

Yield 61%, yellow oil, ^1^H NMR (300 MHz, CDCl_3_): δ 8.26–8.18 (dd, *J* = 0.7 and 7.7 Hz, 1H), 7.66–7.58 (m, 1H), 7.48–7.38 (m, 2H), 7.18–7.10 (t, *J* = 7.9 Hz, 1H), 6.54–6.48 (m, 1H), 6.46–6.36 (m, 2H), 4.24–4.18 (t, *J* = 6.6 Hz, 2H) 3.78 (s, 3H), 3.16–3.08 (t, *J* = 4.61 Hz, 4H), 2.72–2.62 (m, 6H) 1.63 (s, 6H); ^13 ^C NMR (75 MHz, CDCl_3_): δ 177.0, 164.1, 160.5, 152.7, 145.0, 133.9, 129.7, 128.8, 127.2, 125.0, 123.9, 108.6, 104.2, 102.2, 55.5, 55.1, 53.2 (2 C), 49.0 (2 C), 43.5, 37.2, 29.3 (2 C); Formula: C_24_H_29_N_3_O_3_; ESI-MS: 408 [M + H]^+^.

### General procedure for the synthesis of 2–(2-(4–(3-hydroxyphenyl)piperazin-1-yl)ethyl)-4,4-dimethylisoquinoline-1,3(2H,4H)-dione (7)

To a solution of 2–(2-(4–(3-methoxyphenyl)piperazin-1-yl)ethyl)-4,4-dimethylisoquinoline-1,3(2*H*,4*H*)-dione (**6**) (0.312 mmol) in 5 ml of DCM, at 0 °C BBr_3_ (0.624 mmol) was added dropwise. The resulted orange slurry was stirred for 24 h at room temperature. After that time, methanol (10 ml) was added and the reaction mixture was quenched with water. Next, the organic layer was washed with water, dryed over sodium sulfate and the solvent was evaporated. The crude mixture was purified via column chromatography using n-hexane:EtOAc:DCM:MeOH 10:10:79.8:0.2 as eluent.

Yield 75%, brown oil, ^1^H NMR (300 MHz, CDCl_3_): δ 8.25–8.19 (dd, 1H, *J* = 1.0 and 7.7 Hz), 7.66–7.59 (m, 1H), 7.49–7.35 (m, 2H), 7.08–6.98 (m, 1H), 6.49–6.40 (m, 1H), 6.35–6.25 (m, 2H), 5.2 (s, 1H), 4.25–4.19 (t, 2H, *J* = 6.6 Hz), 3.05–3.12 (t, 4H, *J* = 4.4 Hz), 2.74–2.65 (m, 6H), 1.63 (s, 6H); ^13 ^C NMR (75 MHz, CDCl_3_): δ 177.2, 164.2, 152.6, 145.1, 135.8, 133.9, 129.9, 128.9, 127.3, 125.1, 124.7, 108.2, 106.8, 103.1, 55.5, 53.1 (2 C), 48.8 (2 C), 43.6, 37.2, 29.3 (2 C); Formula: C_23_H_27_N_3_O_3_; ESI-MS: 394 [M + H]^+^.

### General procedure for the synthesis of the final molecules (8–10)

#### Method B (for compounds 8–10)

A mixture of 2–(2-chloroethyl)-4,4-dimethylisoquinoline-1,3(2*H*,4*H*)-dione (**2**) (0.247 mmol) and corresponding piperazine (0.5 mmol) was stirred at 140 °C for 30 min. After this time, the reaction mixture was cooled to room temperature, EtOAc (4 ml) was added, and the resulted solid was filtered off. The crude mixture was purified via column chromatography using n-hexane:Et_2_O:DCM 20:40:40 or DCM:EtOAc:MeOH 69.8:30:0.2 as eluent.

### 4,4-Dimethyl-2–(2-(4-(pyrimidin-2-yl)piperazin-1-yl)ethyl)isoquinoline-1,3(2H,4H)-dione (8)

Yield 30%, dark yellow oil, ^1^H NMR (300 MHz, CDCl_3_): δ 8.26–8.18 (dd, *J* = 0.7 and 7.7 Hz, 1H), 7.63–7.60 (m, 1H), 7.48–7.39 (m, 2H), 7.09–6.99 (m, 2H), 6.48–6.44 (m, 1H), 4.25–4.18 (t, *J* = 6.6 Hz, 2H) 3.77, 3.16–3.07 (t, *J* = 4.6 Hz, 4H), 2.73–2.61 (m, 6H) 1.63 (s, 6H); ^13 ^C NMR (75 MHz, CDCl_3_): δ 178.1, 165.0, 147.8, 145.0, 135.1, 134.2, 129.0, 127.4, 125.1, 124.7, 123.5, 121.1, 61.7 (2 C), 43.7 (2 C), 42.9, 34.2, 30.1, 29.3 (2 C) Formula: C_21_H_25_N_5_O_2_; ESI-MS: 380 [M + H]^+^.

### 2–(2-(4–(2,2-Dimethylbenzo[d][1,3]dioxol-4-yl)piperazin-1-yl)ethyl)-4,4-dimethylisoquinoline-1,3(2H,4H)-dione (9)

Yield 39%, pale yellow oil, ^1^H NMR (300 MHz, CDCl_3_): δ 8.25–8.18 (dd, *J* = 0.7 and 7.7 Hz, 1H) 7.66–7.58 (m, 1H), 7.48–7.39 (m, 2H), 6.74–6.66 (t, *J* = 7.9 Hz, 1H), 6.44–6.33 (m, 2H), 4.25–4.15 (t, *J* = 6.4 Hz, 2H), 3.17–3.08 (m, 4H), 2.75–2.61 (m, 6H), 1.68 (s, 6H), 1.66 (s, 6H); Formula: C_26_H_31_N_3_O_4_; Anal. calcd for C_26_H_31_N_3_O_4_: C, 69.47; H, 6.95; N, 9.35; Found: C, 69.25; H, 6.99; N, 9.38; ESI-MS: 450 [M + H]^+^.

### 4,4-Dimethyl-2–(2-(4–(2-oxo-2,3-dihydrobenzo[d]oxazol-7-yl)piperazin-1-yl)ethyl)isoquinoline-1,3(2H,4H)-dione (10)

Yield 33%, yellow oil, ^1^H NMR (300 MHz, CDCl_3_): δ 8.26–8.19 (dd, *J* = 0.7 and 7.6 Hz, 1H) 7.67–7.59 (m, 1H), 7.50–7.33 (m, 3H), 7.08–6.96 (m, 1H), 6.64–6.54 (m, 2H), 4.24–4.18 (t, *J* = 6.6 Hz, 2H), 3.30–3.20 (t, *J* = 4.3 Hz, 4H), 2.78–2.67 (m, 6H), 1.63 (s, 6H); Formula: C_24_H_26_N_4_O_4_; Anal. calcd for C_24_H_26_N_4_O_4_: C, 66.34; H, 6.03; N, 12.89; Found: C, 66.28; H, 6.07; N, 12.94; ESI-MS: 435 [M + H]^+^.

### 4,4-Dimethyl-2–(2-(4–(3-oxo-3,4-dihydro-2H-benzo[b][1,4]oxazin-8-yl)piperazin-1-yl)ethyl)isoquinoline-1,3(2H,4H)-dione (11)

Yield 67%, yellow oil, ^1^H NMR (300 MHz, CDCl_3_): δ 8.26–8.19 (dd, *J* = 0.7 and 7.7 Hz, 1H) 7.66–7.59 (m, 1H), 7.49–7.35 (m, 2H), 7.00–6.98 (m, 1H), 6.90–6.83 (t, *J* = 8.2 Hz, 1H), 6.62–6.58 (dd, *J* = 2.3 and 6.9 Hz, 1H), 6.45–6.49 (d, *J* = 7.6 Hz, 1H), 4.60 (s, 2H), 4.25–4.19 (t, *J* = 6.6 Hz, 2H), 3.08–3.00 (m, 4H), 2.78–2.64 (m, 6H), 1.63 (s, 6H); Formula: C_23_H_22_N_4_O_4_; Anal. calcd for C_25_H_28_N_4_O_4_: C, 66.95; H, 6.29; N, 12.49; Found: C, 66.87; H, 6.30; N, 12.52; ESI-MS: 449 [M + H]^+^.

### Determination of the intrinsic activity of the test compounds at the α2A-adrenoreceptors and α2B-adrenoreceptors

An intrinsic activity assay was performed according to the instructions of the manufacturer of the assay kit containing ready-to-use cells with stable expression of the α2A-adrenoceptor (In-vitrogen, Life Technologies, Carlsbad, CA, USA) or α2B-adrenoceptor (PerkinElmer, Inc, Waltham, MA, USA).

### Determination of the affinity of the test compounds at the a1-adrenoreceptors and a2-adrenoreceptors

The affinity of the obtained compounds was evaluated by radioligand binding assays (the ability to displace [3H] prazosin and [3H]clonidine from a1- and a2-AR, respectively) on rat cerebral cortex. The brains are homogenised in 20 volumes of an ice-cold 50 mM Tris–HCl buffer (pH 7.6) and is centrifuged at 20,000 g for 20 min (0–4 °C). The cell pellet is resus-pended in the Tris–HCl buffer and centrifuged again. Radioligand binding assays are performed in plates (MultiScreen/Millipore). The final incubation mixture (final volume 300 μL) consisted of 240 ll of the membrane suspension, 30 μL of [3H]prazosin (0.2 nM) or [3H]clonidine (2 nM) solution and 30 μL of the buffer containing seven to eight concentrations (1.0^−11^ to 1.0^−4 ^M) of the tested compounds. For measuring the unspecific binding, phentolamine, 10 lM (in the case of [3H]prazosin) and clonidine, 10 μM (in the case of [3H]clonidine) are applied. The incubation is terminated by rapid filtration over glass fiber filters (Whatman GF/C) using a vacuum manifold (Millipore). The filters are then washed twice with the assay buffer and placed in scintillation vials with a liquid scintillation cocktail. Radioactivity was measured in a WALLAC 1409 DSA liquid scintillation counter. All the assays were performed in duplicate.

### *In vitro* whole blood aggregation test

*In vitro* aggregation tests were conducted using freshly collected whole blood with Multiplate platelet function analyzer (Roche Diagnostics Polska Sp. z o.o., Warsaw, Poland), the five-channel aggregometer based on measurements of electric impedance. The Multiplate analyzer allows the duplicate measurement with dual electrode probes. Blood was drawn from carotid of rats with hirudin blood tube (Roche Diagnostic). 300 μL of hirudin anticoagulated blood was mixed with 300 μL pre-warmed isotonic saline solution containing studied compound in DMSO or DMSO (0.1% final) and pre-incubated for 3 min at 37 °C with continuous stirring. The agonists (ADPtest, COLtest, Roche Diagnostic) were diluted using isotonic sterile NaCl solution. Aggregation was induced by adding collagen (final concentration 1.6 µg/mL), or adrenaline and subthreshod concentration of ADP (final concentration 50 µM + 1.6 µM). Activated platelet function was recorded for 6 min. The Multiplate software analyzed the area under the curve of the clotting process of each measurement and calculated the mean values.

Data were presented as Mean ± SEM. Statistical comparisons were made by the analysis of variance (ANOVA) and significance of the differences between control group and treated groups was determined by Dunnet *post hoc* test. *p* < .05 was considered significant.

### The bioavailability assays

The *in vitro* bioavailability assays were performed by Eurofins Panlabs Inc. (St Charles, USA) according to the methods reported in publications listed below:

Solubility: Lipinski, C.A et al. (2001) Adv Drug Del Rev, 46:3–26^32^,

Protein binding: Banker, M.J et al. (2003) J. Pharm. Sci., 92:967–974^33^,

Caco-2 permeability: Hidalgo, I.J et al. (1989) Gastroenterology, 96:736–749^34^,

Microsomal stability: Obach, R.S et al. (1997) J Pharmacol Exp Ther, 283:4658^35^.

## Results and discussion

### Chemistry

**Scheme 1.**
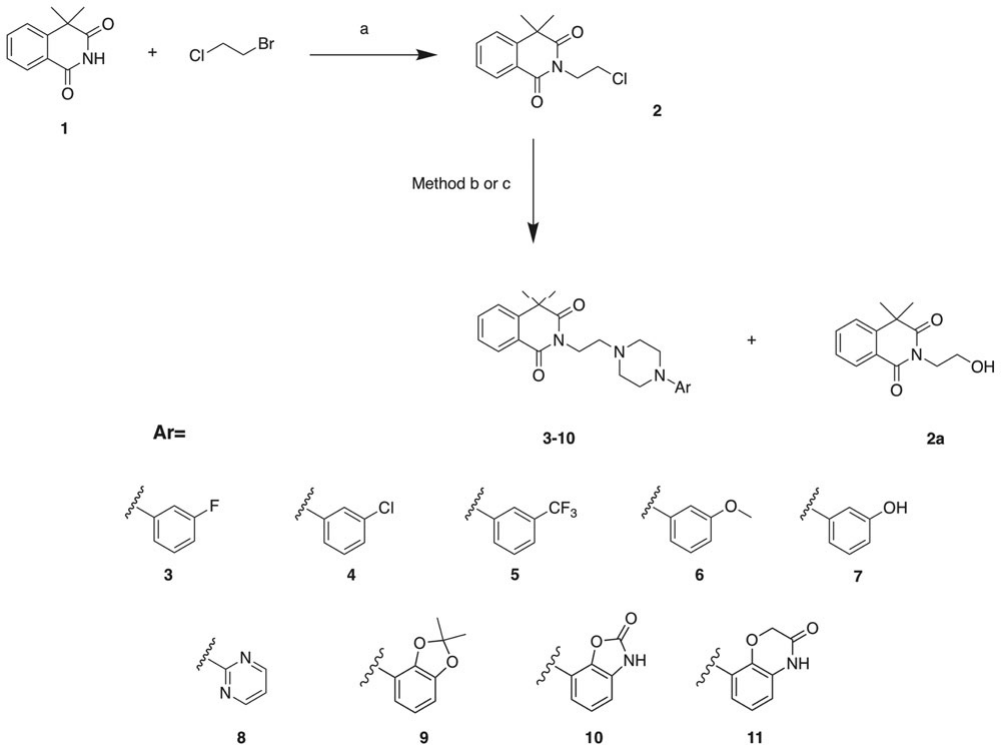
Synthesis of compounds **3**–**11**. Reagents and conditions: (a) TEA, K_2_CO_3_, acetone, reflux, 72 h, 60%; Method b: corresponding arylpiperazine, KI, K_2_CO_3_, dioxane, reflux, 72 h; Method c: corresponding arylpiperazine, 140 °C, 30 min. Demethylation of **6**: BBr_3_, DCM, 0 °C, 24 h.

The synthesis of a series of *N*-arylpiperazine derivatives of 4,4-dimethylisoquinoline-1,3(2*H*,4*H*)-dione (**3–11**) is presented in the Scheme 1. In the first step, alkylation of commercially available 4,4-dimethylisoquinoline-1,3(2*H*,4*H*)-dione (**1**) with 1-chloro-2-bromoetane in the presence of potassium carbonate and triethylamine yielded 2–(2-chloroethyl)-4,4-dimethylisoquinoline-1,3(2*H*,4*H*)-dione (**2**). The intermediate (**2**) was next reacted with corresponding arylpiperazines to give the final compounds **3–11**. Initially, we obtained the final molecules **3–11** with relatively small insufficient yields (8–15%), which was related to the parallel formation of the side product (**2a**). Therefore, we optimized the reaction conditions, changing the solvent from acetonitrile to dry dioxane, which allowed us to obtain the final products **8–11** with higher yields (30–67%, Method b, Scheme 1). Compounds **3–6** were obtained in solvent-free conditions, reacting the intermediate (**2**) with an excess of corresponding arylpiperazines (Method c, Scheme 1), which afforded the final compounds **3–6** with satisfactory yields (35–67%). Additionally, compound **7** was obtained via demethylation of corresponding methoxy derivative **6**, using BBr_3_ in DCM at 0 °C.

## Molecular properties and predicted ADMET parameters

The newly designed structures were tested for compliance with two rules determining drug-like properties. Lipinski rule of five and Veber filter evaluate bioavailability of a compound after oral administration. The first one assumes that compounds having LogPo/w (octanol/water partition coefficient) lower than 5, MW (molecular weight) below 500, less than 10 HBA (H-bond acceptors), and 5 HBD (H-bond donors) are more likely to show favorable bioavailability^32^. The Veber rule extends range of parameters with <10 rotatable bonds and TPSA (total polar surface area) of <140 A^2 36^. Molecules that obey the restrictions are more likely to show preferable membrane permeability. To this end, molecular properties of the studied compounds were calculated (Table 1).

Properties calculated using QikProp software (Schrödinger Ltd): QPLogP – Predicted octanol/water partition coefficient; MW – molecular weight; HBD – hydrogen bond donor; HBA – hydrogen bond acceptor; RB – rotatable bonds; TPSA – total polar surface area (A^2^). Number of PAINS alerts reported by SwissADME.

The novel compounds comply with Lipinski and Veber rules and may be therefore considered drug-like. The determined crucial molecular properties show high probability that the molecules will be bioavailable *per os*. Moreover, the designed structures were examined for known classes of reactive assay interference compounds that would disturb biological *in vitro* studies. According to SwissADME tool^31^, none of the compounds contain substructural features recognized as pan assay interference compounds (PAINS) (Table 1).

To further characterize the designed molecules, important ADME parameters were predicted. The compounds were characterized by moderate to high predicted water solubility (20–1259 µM/L, expect of compound **5** – 1 µM/L), which together with fair predicted Caco-2 cells permeability (compounds having permeability values over 500 nm/s are considered well-permeable through gut-blood barrier) stands for their favorable predicted human oral absorption (78–100%). Majority of the compounds (except compounds **8**, **10,** and **11**) were predicted to have the ability to cross the blood-brain-barrier (BBB). It has been suggested that anticoagulant activity in central nervous system might be regarded as potential prevention of brain stroke, thus the ability of designed compound to cross the blood-brain-barrier in this aspect might be beneficial^37^. Moreover, compounds **3**–**6** are not supposed to be P-gp substrates. The tested compounds are expected to bind with serum albumins at the rate of 69–100% and are supposed to have fair metabolic stability after CYP3A4 incubation (16–45% compound remaining, while compounds having predicted over 50% are considered metabolically stable) (Table 2).

**Table 2.** Predicted ADME parameters.

Compound	QPlogS	QPPCaco [nm/s]	PO [%]	BBB	Pgp	PB [%]	MetStab [%]
**3**	−4.5	927	100	Yes	No	87	29
**4**	−4.7	850	100	Yes	No	100	23
**5**	−6.0	676	94	Yes	No	92	19
**6**	−4.2	688	100	Yes	No	88	27
**7**	−4.2	231	89	Yes	Yes	84	36
**8**	−2.9	486	91	No	Yes	69	45
**9**	−4.7	707	100	Yes	Yes	90	16
**10**	−3.3	122	78	No	Yes	81	42
**11**	−4.4	120	80	No	Yes	84	36

Predicted parameters: QPlogS – solubility; QPPCaco – Caco-2 cell permeability; %PO Absorption – percent human oral absorption (QikProp, Schrödinger Ltd.); BBB – blood-brain-barrier permeability; Pgp – substrate of glycoprotein P (SwissADME); PB – % of protein binding; MetStab – metabolic stability after CYP incubation (VolSurf+, Molecular Discovery).

### *In vitro* assays

Considering that a potent blockade of alpha 2B-ARs is required for the antiplatelet effect^8^ ^,^ ^9^ we began an assessment of a pharmacological profile of all the synthesized molecules (**3–11**) with the evaluation of their alpha 2B-ARs antagonistic properties. The majority of the final molecules (**3**, **4**, **9,** and **10**) elicited a potent blockade of the alpha 2B-ARs, with the IC_50_ values ranging from 47 to 251 nM (Table 3). Next, we have determined the selectivity of the obtained compounds vs. alpha 2 A-adrenoreceptor subtype. All of the molecules showed a negligible affinity for alpha-2 A adrenoreceptor giving no significant effect at the concentration of 1.0E–05 M. The above results suggest the desired level of selectivity vs. alpha 2 A-AR subtype.

**Table 3.** Functional activity results for compounds **3**–**11**. Antagonist potency towards alpha 2B-AR, expressed as IC_50_ (nM) ± SEM values

Compound	Antagonist mode (IC_50_ ± SEM) [nM]
**3**	61 ± 25.4
**4**	251 ± 71
**5**	>1000
**6**	>1000
**7**	758 ± 160
**8**	>1000
**9**	47 ± 12.8
**10**	61 ± 22.5
**11**	>1000
**ARC-239**	8.4 ± 3.2

Subsequently, we determined the selectivity vs. alpha 1-adrenoreceptor (Table 4). The final compounds **3–11** were submitted to a radioligand binding assay, measuring the ability to displace [^3^H] prazosin from alpha 1-ARs, in the rat cerebral cortex. ARC-239 was used as a reference and it showed high binding properties for alpha 1-ARs, with the K_i_ value of 0.3 nM. These results are with the agreement with the previous reports^13^ ^,^ ^15^. The majority of newly synthesized compounds showed a weaker affinity for alpha 1-ARs comparing to ARC-239. It was found that the replacement of 2-methoxybenzene group with pyrimidine ring caused the most significant drop in alpha1-AR affinity. On the other hand, the introduction of a hydrophobic group into meta position of the phenylpiperazine ring (**3, 4, 5, 6**) caused a relatively weaker decrease. Interestingly, incorporation of hydroxyl group into meta position of the phenylpiperazine ring gave similar result and caused slight decrease in affinity. The incorporation of a bulky substituents such as; 2,2-dimethylbenzo[*d*][1,3]dioxole (**9**), benzo[*d*]oxazol-2(3*H*)-one (**10**), 2*H*-benzo[*b*][1,4]oxazin-3(4*H*)-one (**11**) maintained the affinity for alpha1-ARs. However, the K_i_ (3–30 nM) values were still significantly higher than for ARC-239. The results of structure-activity relationship unambiguously showed that the introduced modifications maintained antagonistic activity at alpha 2B-AR, did not increase the affinity for alpha 2 A-AR and reduced the affinity for alpha 1-AR.

**Table 4.** The results of binding to alpha1-AR of the final compounds **3**–**11** and the reference ARC-239 expressed as K_i_ ± SD values.

Compound	Affinity for alpha 1-ARs K_i_ ± SD [nM]
**3**	30.0 ± 1.0
**4**	93.0 ± 2.0
**5**	703 ± 1
**6**	78.4 ± 3.8
**7**	81.70 ± 5.5
**8**	1500.0 ± 100.0
**9**	30 ± 0.2
**10**	10.3 ± 0.3
**11**	3.0 ± 0.1
**ARC-239**	0.3

Based on the aforementioned results, the most interesting compounds (**3**, **4**, **9,** and **10**) were selected for further studies. In order to evaluate the anti-platelet effects of the new compounds *in vitro*, freshly isolated rat whole blood was incubated with selected compounds (3–100 μM) or vehicle (DMSO), and the aggregation responses were evaluated with multiplate whole blood aggregometer by measuring impedance change. Platelet aggregation was induced by collagen or sub-threshold concentration of ADP and adrenaline. ARC-239 was used as a reference compound.

**Figure 3.**
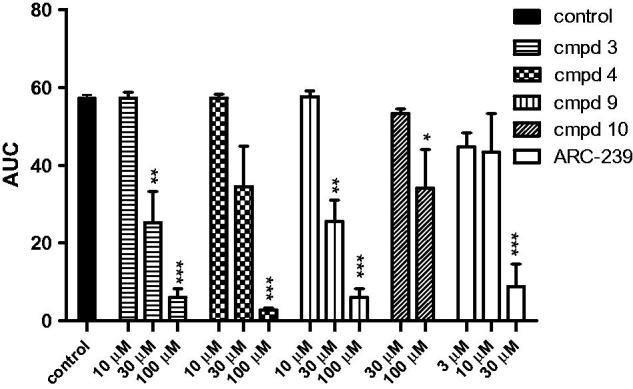
Effects of the studied compounds and ARC-239 on *in vitro* whole rat blood aggregation induced by collagen (1.6 µg/mL). Results are expressed as mean ± SEM, *n* = 3–6, *p < .05, **p < .01, ***p < .001 vs. control group (0.1% DMSO in saline).

Compounds **3**, **4**, **9,** and **10** were found to inhibit collagen-induced platelet aggregation *in vitro* as presented in Figure 3 and Table 3. Compound **9** was active at the concentration of 100 μM, attenuating platelet aggregation to 59.3%. Compounds **3**, **4,** and **9** exhibited significant anti-platelet efficacies also at lower concentration (30 μM) giving IC_50_ values ranging from 26.9 ± 2.5 μM^3^ to 34.5 ± 18.8 μM^4^. The IC_50_ value for ARC-239 was in the similar range as for the studied compounds and was equal to 20.7 ± 14.7 μM.

**Figure 4.**
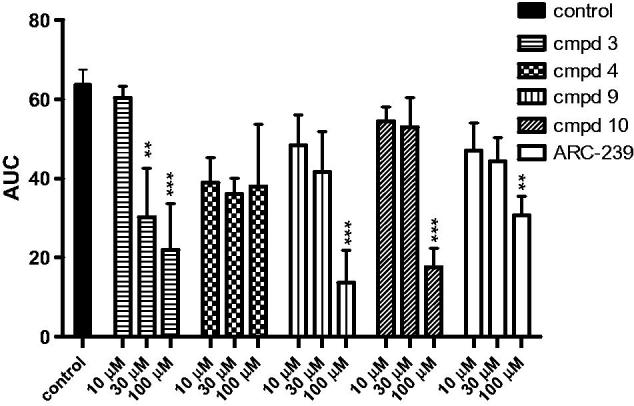
Effects of studied compounds and ARC-239 on *in vitro* whole rat blood aggregation induced by simultaneous addition of adrenaline and ADP (50 µM + 1.6 µM). Results are expressed as mean ± SEM, *n* = 3–9, **p < .01, ***p < .001 versus control group (0.1% DMSO in saline).

Further studies showed that three compounds: **3**, **9,** and **10** also inhibited aggregation induced by the sub-threshold concentration of ADP and adrenaline. At a concentration of 1.6 μM, ADP alone, only partially and transiently aggregated rat blood *in vitro*, whereas adrenaline alone did not cause aggregation at any concentration tested. Combining adrenaline with the sub-threshold concentration of ADP produced a maximal aggregation response. The adrenaline-mediated amplification of ADP-stimulated aggregation was attenuated when rat blood was pre-incubated with **3**, **9**, **10,** and ARC-239. The IC_50_ values ranged from 20.5 μM^3^ to 76.5 μM^10^. The IC_50_ value for ARC-239 was in the similar range as for the studied compounds **9** and **10** and it was equal to 63.9 ± 21.3 μM. Compound **4**, even up to 100 μM, did not exhibit any significant inhibition against ADP and adrenaline induced blood aggregation. The results are presented in Figure 4 and Table 5. Compound **3** was observed to be the most potent among the entire series and exhibited an IC_50_ of 26.9 μM against collagen and 20.5 μM against ADP and adrenaline induced blood aggregation and **3** was also superior to ARC-239 concerning ADP-adrenaline induced aggregation.

**Table 5.** Potencies of the studied compounds and ARC-239 in inhibition *in vitro* whole rat blood aggregation induced by (A) collagen (1.6 µg/mL), (B) ADP and adrenaline (1.6 µM + 50 µM).

Compound	A (collagen) IC_50_ [μM]	B (ADP + A) IC_50_ [μM]
**3**	26.9 ± 18.8	20.5 ± 7.1
**4**	34.5 ± 2.5	n.a.
**9**	27.4 ± 5.3	54.4 ± 5.4
**10**	n.a.	76.5 ± 5.8
**ARC-239**	20.7 ± 14.7	63.9 ± 21.3

IC_50_ (concentration of the compound that inhibits the whole rat blood aggregation *in vitro* by 50%), n.a. – not active.

Concerning the described above results, for the most promising compound **3,** we performed early *in vitro* bioavailability assays, including aqueous solubility, human plasma protein binding, human liver microsomes stability, and Caco-2 permeability (Eurofins Bioavailability panel). The results are summarized in Table 6.

**Table 6.** *In vitro* bioavailability data for compound **3**.

Assay	Test Concentration [M]	Property			
		Solubility [µM]			
Aqueous solubility (simulated intestinal fluid)	2.0E-04	68.0			
Aqueous solubility (PBS, pH 7.4)		5.4			
Aqueous solubility (simulated gastric fluid)		150.9			
		% Protein bound			
Protein binding (plasma, human)	1.0E-05	99			
		Permeability [10^−6^ cm/s]			
A-B permeability (Caco-2, pH 6.5/7.4)	1.0E-05	7.7			
B-A permeability (Caco-2, pH 6.5/7.4)		2.6			
		Incubation time [minutes]	% Compound remaining	Half-life [minute]	Intrinsic clearance [μL/min/mg]
				14.1	14
Metabolic stability (liver microsomes, human)	1.0E-07	0	100.0		
		15	49		
		30	18		
		45	8		
		60	6		

Compound 3 displayed moderate aqueous solubility (in PBS pH 7.4 = 5.4 µM, simulated gastric fluid =150.9 µM and simulated intestinal fluid =68.0 µM), high plasma protein binding (99%), fair metabolic stability (half-life 14.1 min, intrinsic clearance 14 ml/min/mg), and fair Caco-2 permeability (7.7 x 10^–6^ cm/s). Such characteristics leave space for further optimization; however, they support the selection of compound **3** for subsequent *in vivo* studies, that will be addressed in the future.

## Conclusions

In summary, we have synthesized a series of *N*-arylpiperazine derivatives of 4,4-dimethylisoquinoline-1,3(2*H*,4*H*)-dione as potent alpha 2B-receptor antagonists. The compounds were generated by changing the substitution pattern at the phenylpiperazine moiety of a known alpha 2B ARs antagonist, compound ARC-239, which also exhibits a strong binding affinity for alpha 1 AR receptors. The applied modification maintained an antagonistic activity at alpha 2B-ARs and reduced the affinity for alpha 1-ARs. The anti-platelet effects of the new compounds were evaluated in *in vitro* models. The most potent analog among all the series was compound **3**, since it effectively inhibited the platelet-aggregation induced both by collagen and ADP/adrenaline. At the same time, compound **3** displayed drug-like properties in computational predictions, which were positively verified by *in vitro* bioavailability assays. The results of our study confirm that the alpha 2B-AR antagonists remain an interesting target for the development of novel antiplatelet agents with a different mechanism of action. Further studies to extend the pharmacological profile of obtained compounds will be conducted.
